# IFI6 Inhibits Apoptosis via Mitochondrial-Dependent Pathway in Dengue Virus 2 Infected Vascular Endothelial Cells

**DOI:** 10.1371/journal.pone.0132743

**Published:** 2015-08-05

**Authors:** Yiming Qi, Ying Li, Yingke Zhang, Lin Zhang, Zilian Wang, Xuzhi Zhang, Lian Gui, Junqi Huang

**Affiliations:** 1 Guangdong Provincial Key Laboratory of Organ Donation and Transplant Immunology, Guangzhou, PR China; 2 First Affiliated Hospital, Sun Yat-sen University, Guangzhou, China; 3 Institute of Immunology, Zhongshan School of Medicine, Sun Yat-sen University, Guangzhou, PR China; 4 Key Laboratory of Tropical Diseases Control, Ministry of Education, Guangzhou, PR China; University of Rochester, UNITED STATES

## Abstract

Dengue hemorrhagic fever (DHF)/Dengue shock syndrome (DSS) is a fatal infectious disease that demands an effective treatment. Interferon (IFN)-stimulated genes (ISGs) induced by dengue virus (DENV) exert antiviral effects. Among ISGs, IFN-α inducible gene 6 (IFI6) was increased in DENV infected human umbilical vascular endothelial cells (HUVECs) by microarray analysis in our previous study. However, its function is incompletely understood. In this study, we confirmed that IFI6 was markedly induced in DENV infection of both primary HUVECs and EA.hy926 cell lines. Recombinant EA.hy926 cell lines in which IFI6 was either over-expressed (IFI6+/+) or knocked-down (IFI6-/-) were generated. The activation of caspase-3 and intrinsic apoptosis-related protein caspase-9 were down-regulated in IFI6+/+ but up-regulated in IFI6-/- cells at 24–48 hrs post-infection. After incubation with DENV for 48 hrs, the mitochondrial membrane potential (Δψ(m)) was more stable in IFI6+/+ cells but reduced in IFI6-/- cells, as assayed by fluorescence staining with JC-1. We observed that Bcl-2 expression was increased in IFI6+/+ and decreased in IFI6-/- cells. By contrast, Bax expression was decreased in IFI6+/+ and increased in IFI6-/- cells. It is presumed that the anti-apoptotic function of IFI6 is expressed by regulating the rheostatic balance between bcl-2/bax expression and inhibition of Δψ(m) depolarization during DENV infection of vascular endothelial cells(VECs). In addition, the pro-apoptotic protein X-linked Inhibitor of Apoptosis (XIAP)-Associated Factor 1(XAF1) expression had been reported to be up-regulated and led to the induction of apoptosis in DENV2-infected VECs,but the relationship between XAF1 and IFI6 dengue virus-induced apoptosis in VECs warrants further study.

## Introduction

Dengue virus (DENV), which belongs to the flavivirus family, has four serotypes including DEN1 to DEN4. It is one of the most prevalent mosquito-borne viruses that cause roughly 390 million infection in humans annually [1]. Unfortunately, there is neither effective therapeutics nor approved vaccines available to prevent or treat infection [2]. Therefore, detailed understanding of the immunopathology of the interaction of DENV with the host is an urgent research need, which should contribute important knowledge in the exploration of new ways to combat this tropical communicable disease.

Clinical manifestation of DENV infection is highly variable, and ranges from unapparent infection to a mild febrile syndrome or even fatal disease with a mortality rate of 5% to 30% [3]. Although a majority of DENV infections display a self-limited course, which are resolved in several days, there remain some susceptible patients that would progress to dengue hemorrhagic fever (DHF), which is characterized by increasing vascular permeability and plasma leakage that manifests in approximately three days following infection [4]. In rare cases, a more sudden onset and extensive syndrome, might progress to shock or even death, which is referred to as dengue shock syndrome (DSS). Usually, the hinge point of the disease always appears following a temporal defervescence phase, after which patients either gradually improve or progressively decline to more severe DHF and DSS. Thus, endothelial cells are regarded as the primary cellular barrier and the major defense system of the vasculature to resist viral infection and in the determination of the prognosis of disease [5]. However, the potential mechanisms of endothelial cells during DENV infection, especially at early stages of disease, currently remain obscure.

Type I interferon (IFNα/β) is produced by most cells, and plays a crucial role in the ability of the endothelium to withstand DENV infection. Increases in gene expression and circulating levels of type I IFN were confirmed by many studies [6, 7], and type I IFN is highly effective at inhibiting DENV replication during early short periods of viremia in most mild cases. However, in severe forms of DENV replication, the ability of type I IFN to inhibit viral replication is overwhelmed [8].

The canonical pathway of Type I IFN is the IFN-stimulated gene factor (ISGF)-3 pathway, which is formed by IFN-regulatory factor (IRF)-9, phosphorylation of signal transducer and activator of transcription (STAT)-1/STAT-2 and sequential activation of the IFN-stimulated response element (ISRE) that leads to transcriptional up-regulation of many IFN-stimulated genes (ISGs) [9]. IFNα inducible protein 6 (IFI6), previously known as G1P3, IFI-6-16 and IFI616, is a type I ISG that is located on chromosome 1p35 [10], and alternatively spliced transcript variants from exons two and three that encode different isoforms of IFI6 by using two downstream repeat units as splice donor sites enable transcription of three mRNA species [11]. Predominant production of IFI6 is the 14 kDa hydrophobic protein form of 130 amino acids, which may play a central role in regulating apoptosis and immunomodulation [12]. Additionally, the Janus tyrosine kinase (JAK)/ STAT signal transduction pathway regulates IFI6, which is a mitochondria-targeted protein that blocks the release of cytochrome c from mitochondria, and thus delays programmed cell death (apoptosis) that is initiated and signaled by the tumor necrosis factor (TNF)-related apoptosis-inducing ligand (TRAIL)/ caspase-8 pathway [13, 14]. IFI6 is thus widely considered a pro-survival factor [15]. Although the functional role of IFI6 in the immune system is quite well known, its antiviral effects are poorly understood [16].

Microarray studies have assisted our understanding of the different gene expression profiles seen in DENV infected VECs[17, 18]. A series of IFN-stimulated and IFN-inducible genes are highly expressed post-infection, and include ISG15, IFI6, IFI44, IFI44L, IRF27, IFIT1, IFIT3, and XAF1. Among these, IFI6 is not only one of the most upregulated genes [19] but is also a specific target for yellow fever virus (YFV), which is congeneric with DENV [20]. Further, IFI6 belongs to the ISG12 gene family, which is composed of four members, ISG12a, ISG12b, ISG12c and IFI6 [21]. Although they are homologous, the biological functions of the ISG12 family are quite varied, and range from preventing host susceptibility to a destructive stimulus to enhancing the sensitivity to DNA damage-induced apoptosis [16].

As a new member of the ISG12 family, most research has focused on studying the IFI6 upstream regulatory region or the function of IFI6 in cancer cells [14, 16]. Moreover, its expression is extremely high in multi-drug resistant cancer cells, suggesting that a close correlation between IFI6 levels and resistance to apoptosis [16]. However, the potential role of IFI6 and its mechanism of action in infectious diseases, especially in viral infections like DENV, remain poorly defined. We hypothesized that the human ISG12 family protein member, IFI6, might be involved in regulating the intrinsic apoptotic pathway in response to DENV infection of VECs. Primary HUVECs isolated from human umbilical veins and EA.hy926 HUVEC cell line were exposed to DENV2 infection and IFI6 expression was detected on mRNA and protein levels. Then over-expression and knock-down plasmids of IFI6 were constructed and transfected into EA.hy926 cells. Annexin V-based flow cytometry assays in different groups were performed to verify the role of IFI6 in DENV-induced apoptosis and underlying mechanisms were investigated.

## Materials and Methods

### 2.1 Cell lines

Primary HUVECs were isolated from human umbilical veins (provided by First Affiliated Hospital, Sun Yat-Sen University, Guangzhou, China) in the study and the study was approved by Medical Ethical Committee of The First Affiliated Hospital, Sun Yat-sen University. Primary HUVECs were cultured in endothelial cell growth medium (30% serum-free medium (SFM) and 60% M199, Gibco, USA) with 10% fetal calf serum (FCS), 100 U penicillin-G, 100 μg/mL streptomycin sulfate, and 30 μg/mL endothelial cell growth supplement (Millipore, USA). Samples were obtained after receiving a written informed consent document for each patient.

The EA.hy926 HUVEC cell line was obtained from the Center for Stem Cell Biology and Tissue Engineering (Sun Yat-Sen University, China). Cells were cultured in DMEM medium (Thermo Scientific, USA) with 10% FCS, 100 U penicillin-G, and 100 μg/mL streptomycin sulfate.

### 2.2 DENV2 preparation and infection

The DENV2 serotype originated from the New Guinea C strain was reservation in our laboratory, which was propagated and quantified as previously described [22]. All infected cells in the experiments were incubated with DENV2 at a multiplicity of infection (MOI) of 4 for 2 hrs and then the suspension medium containing DENV2 particles was removed. The culture medium was replaced with 2% FCS, followed by an additional incubation for up to 48 hrs. Vector cells were processed in parallel as controls.

### 2.3 Identification of EA.hy926 cells

The phenotype of EA.hy926 were confirmed by immunocytochemical staining for factor VIII and surface expression of platelet/endothelial cell adhesion molecule 1 (CD31) was detected. Cells were fixed in 4% paraformaldehyde for 15 min, permeabilized with 0.5% Triton X-100 for 15 min, and then incubated with 3% hydrogen peroxide for 20 min at room temperature (RT) to quench endogenous peroxidase activity. Fixed cells were then blocked with 5% bovine serum albumin (BSA) for 20 min and incubated overnight at 4°C with an antibody against factor VIII-related antigen (Boster, China). Immunolabeled cells were washed thoroughly in phosphate-buffered saline (PBS), followed by incubation with a horseradish peroxidase-conjugated secondary antibody (Abcam, UK) for 1 h at RT. Immunostaining was visualized by incubation in the chromogen diaminobenzidine (DAB) for 5 min at RT. Expression of CD31 was assessed by flow cytometry. Cells were collected and stained with anti-CD31-FITC (Becton Dickinson, US) for 30 min at RT, followed by fixation for 20 min at 4°C. Samples were loaded in a FACSCalibur system (Becton-Dickinson, US) and results analyzed using FlowJo software (TreeStar, San Carlos, CA, USA).

### 2.4 Construction of stable EA.hy926 cell lines by over-expression or knock-down of IFI6

Retroviral-mediated stable expression of IFI6 was achieved by using EA.hy926 cells and the pBabe-Puro Retroviral Vector system (pBabe; Addgene, Cambridge, MA, USA) or the pSUPER RNAi system (pSUPER.retro.puro, pSUPERretro; Addgene) to over-express or knock-down gene expression, respectively.

The over-expression primers were: forward, 5’-CGGAATTCATGCGGCAGAAGGCGGTATC-3’ and reverse, 5’-GAAGATCTCTACTCCTCATCCTCCTCACTAT-3’. The knock-down primers were: forward, 5’-GAGAATGCGGGTAAGGATGCATTCAAGAG-A-3’ and reverse, 5’-GAGAATGCGGGTAAGGATGCATCTCTTGAA-3’. The over-expressed plasmid was named pMSCV-neo-IFI6^+^ with the corresponding vector named vector-high. The knock-down plasmid was named pSUPERretro-IFI6^-^ with the corresponding vector named vector-low. The plasmids were constructed, and then transfected into EA.hy926 cells as described previously [19].

### 2.5 MTT Assay

5000 cells/well were seeded into 96-well culture plates one night before treatment. After that, cells were infected by DENV2 with MOI 4. MTT reagent was added at 20 μL (5 mg/mL) per well at various times, and cells were then incubated for another 4 hrs at 37°C. The reaction was stopped by adding 150 μL dimethyl sulfoxide (DMSO), following which a purple formazan precipitate dissolved in the DMSO. The optical density was measured at a wavelength of 490 nm using an MRX II absorbance reader (BioRad, USA). With the background absorbance subtracted, results were described as mean values, which were measured in triplicate and repeated from three independent experiments.

### 2.6 TUNEL analysis and immunofluorescence

Cells were cultured on glass slides, treated as described above, and then rinsed twice in PBS. Slides were then fixed in 4% paraformaldehyde, and cells were perforated by treating them with 0.5% Triton X-100 at room temperature for 15 min. Next, cells were incubated with 0.125% pamcreatin for another 10 min, and cells were blocked with 5% BSA at room temperature. Redundant fluid was discarded 30 min later, and cells were then incubated with the primary antibody NS1 at a dilution of 1:10 (Abcam, USA) overnight at 4°C. Next, a secondary antibody anti-mouse-Cy3 at a dilution of 1:1000 (Invitrogen, USA) was added at room temperature for 30 min, followed by rinsing in PBS and staining with the Tunel reaction mixture for 60 min, following the manufacturer’s guidelines. Finally, DAPI staining (37°C for 15 min) was used to visualize the nucleus, and observed by confocal microscopy.

### 2.7 Flow cytometry

The frequency of DENV-induced apoptosis in both endothelial cells and recombinant cells was determined with an Annexin V-FITC/PI Apoptosis Detection Kit (KeyGEN, China). Annexin V that had bound to the externalization of phophatidylserine, which is an early apoptotic event of the cell, was analyzed by flow cytometry after incubating with DENV2 (MOI = 4) for 24 hrs, 36 hrs and 48 hrs. Approximately 5×10^5^ cells were resuspended in 5 μL Annexin V binding buffer (10 mmol/L Hepes/NaOH (pH 7.4), 140 mmol/L NaCl, and 2.5 mmol/L CaCl_2_). Using double labeling for Annexin V-FITC and PI, we separated sub-populations of early apoptotic cells (Annexin V+/PI−), late apoptotic or necrotic cells (Annexin V+/PI+), and viable cells (Annexin V−/PI−) from each other.

### 2.8 Mitochondrial Membrane Potential (Δ ψ) analysis

Cells were seeded onto small glass slides (Orange Scientific. E.U). Culture medium was abandoned following treatment with DENV2 for 24 hrs, 36 hrs, and 48 hrs, respectively. JC-1 assay reagent (25 μmol/L) was added to the dilution buffer (500 μL/well), which was then incubated at 37°C for 20 min to stain the mitochondria. After 2 to 3 rinses with wash buffer, cells were inspected using an Axiovert 200 fluorescent inverted microscope(Zeiss, Germany). Both monomeric (excitation at 488nm, emission 500–550nm) as well as aggregation (excitation 488 nm, emission at 575–620nm) were registered using the microscope. In each slide, about 200 cells were counted to make sure the results are visualize and comparable.

### 2.9 Immunoblotting

Treated cells were harvested and lysed using a mammalian protein extraction reagent (Kangwei Company, China). Whole-cell lysates of a fixed quantity (30 μg/lane) were subjected to 10% SDS-PAGE and then transferred to a polyvinyldine fluoride (PVDF) membrane (Millipore, USA). We used the following specific antibodies: anti-IFI6, -caspase-3, -caspase-8, -Bax, and-Bcl-2 (all from Cell Signaling Technology, USA) and anti-XAF1 (Abcam, USA). The antibody dilutions for Western blotting primary antibodies and secondary antibodies were 1:800 and 1:2000, respectively. The vehicles for antibodies were 5% BSA. Enzyme-linked chemiluminescebetance (Kangwei Company, China) was used to detect the target band according to the manufacturer’s instructions. The images were analyzed using the Image J program. Histogram of each image was first extended to saturate the gray scale. Then, the 12-bit images were converted to an 8-bit gray scale. Intensity of each band was divided by the β-actin band firstly, and then the relative value were compared.

### 2.10 Detection of activity of Caspase-9

3–5×10^6^ cells were collected, resuspended in 50 μL of cold cell lysis buffer and incubated on ice for 10 min. Cells were centrifuged for 1 min (10, 000×g). The supernatant (cytosolic extract) was transferred to a new tube and immediately placed on ice. The BCA Protein Assay Kit was used to assay the protein concentration. Each cytosolic extract was diluted to a concentration of 50–200 μg protein per 50 μL of cell lysis buffer. Then DTT was added to the 2×Reaction Buffer immediately before use (10 mmol/L final concentration), and 50 μL of 2×Reaction Buffer (containing 10 mmol/L DTT) was added to each sample. Next, added 5 μL of 4 mmol/L LEHD-pNA substrate (Invitrogen, Shanghai, China) which is composed of the chromophore, p-nitroanilide (pNA), and asynthetic tetrapeptide, LEHD (Leu-Glu-His-Asp),. samples were then incubated in the dark at 37°C for 2 hrs. Samples were read at a wavelength of 400 nm or 405 nm in a microplate reader (BioTek, Vermont, USA).

### 2.11 Statistical analysis

Data are expressed as mean±s.e.m. All data groups were analyzed by Student’s T test with the software program SPSS v. 16.0 (SPSS Inc., Chicago, IL, USA). An alpha value of P<0.05 was used to determine the statistical significance when interpreting the results.

## Results

### 3.1 IFI6 is induced in VECs in response to DENV2 infection

**Fig 1:**
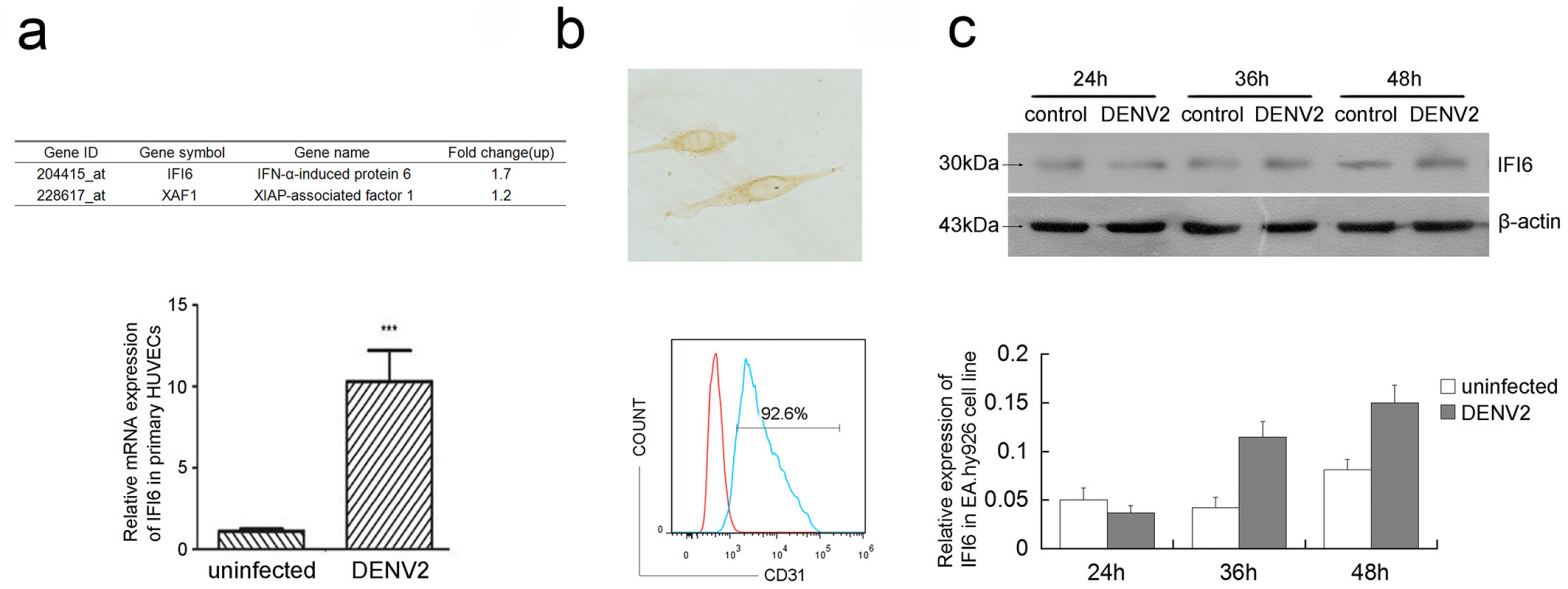
Dengue virus infection induced IFI6 over-expression in isolated primary HUVECs and EA.hy926 cells. Expression of IFI6 was detected by GeneChip hybridization and analyzed(see Panel a, top) and mRNA expression of IFI6 was detected using qRT-PCR (see Panel a, below) at 48 hrs post-infection in HUVECs. Factor VIII antigen staining by immunocytochemistry (×400)(see Panel b, top). Analysis of CD31 expression in EA.hy926 cells by flow cytometry. The red was the negative control(see Panel b, below). (c) Protein expression of IFI6 was detected using immunoblotting in EA.hy926 cells after 24, 36, and 48 hrs post DENV2 infection. The gray scale scanning data were shown below and normalized to β-actin.

*IFI6* up-regulated in HUVECs after infection with DENV2 for 48 hrs by GeneChip hybridization and analysis (Fig 1, panel a, top), and mRNA expression of IFI6 in primary HUVECs isolated from human umbilical veins was markedly up-regulated after 48 hrs post DENV2 infection(Fig 1, panel a, below). EA.hy926 immunostained with the antibody against factor VIII and an HRP-conjugated secondary antibody exhibited brown DAB reaction product (Fig 1, panel b, top), while EA.hy926 cells exhibited CD31 surface staining (Fig 1, panel b, below). Immunobloting assays were employed to validate IFI6 protein levels in EA.hy926 HUVECs cells, confirming that IFI6 was increased following DENV2 infection (Fig 1c).

### 3.2 Generation of EA.hy926 cell lines with IFI6 over-expression or knock-down

**Fig 2:**
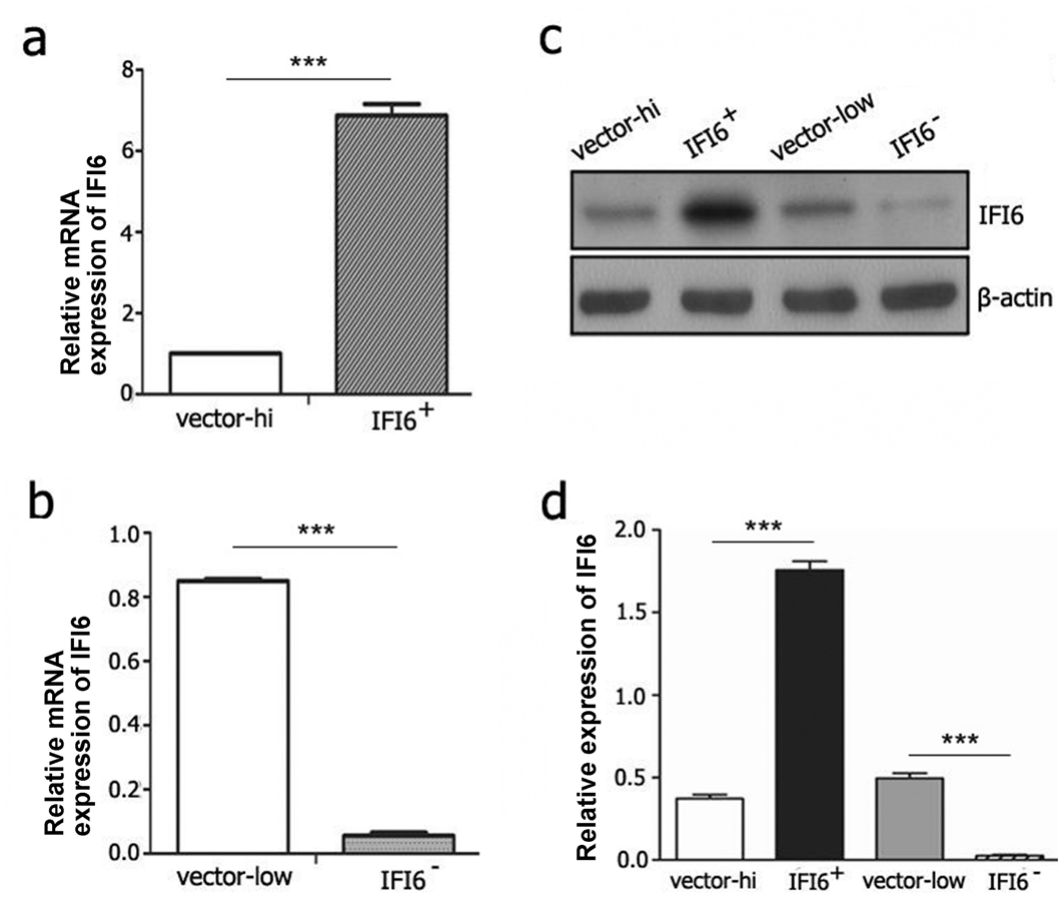
Identification of recombinant vascular endothelial EA.hy926 cell lines with stable over-expression/knock-down of IFI6. (a and b) mRNA expression of IFI6 was detected using qRT-PCR in EA.hy926 cells with vector-hi(a), vector-low(b) and the recombinant cell lines IFI6+/+ (a) and IFI6-/- (b). (c and d) Protein expression of IFI6 was detected using immunoblotting in the indicated lysate (c) and the gray scale scanning data were shown below and normalized to β-actin (d). ***P<0.01.

To further understand the role of IFI6 in apoptosis that was induced by DENV2-mediated infection of VECs, we constructed two stable cell lines of EA.hy926 IFI6+/+ and IFI6-/- cells, which were transfected with IFI6 over-expression (pMSCV-neo-IFI6+) and IFI6 knock-down (pMSCV-neo-IFI6-) plasmids. Empty vectors pMSCV-neo and pSUPERretro transfected cell lines were also generated as controls, which were referred to as “vector-high” and “vector-low”, respectively. Next, cells were subjected to qRT-PCR analysis of total RNA extracts and immunobloting of protein lysates in order to confirm alteration of IFI6 expression. The results were shown in Fig 2. Both mRNA and protein levels of IFI6 were significantly increased in IFI6+/+ cells and decreased in IFI6-/- cells.

### 3.3 IFI6 suppresses DENV2-induced apoptosis in VECs

**Fig 3:**
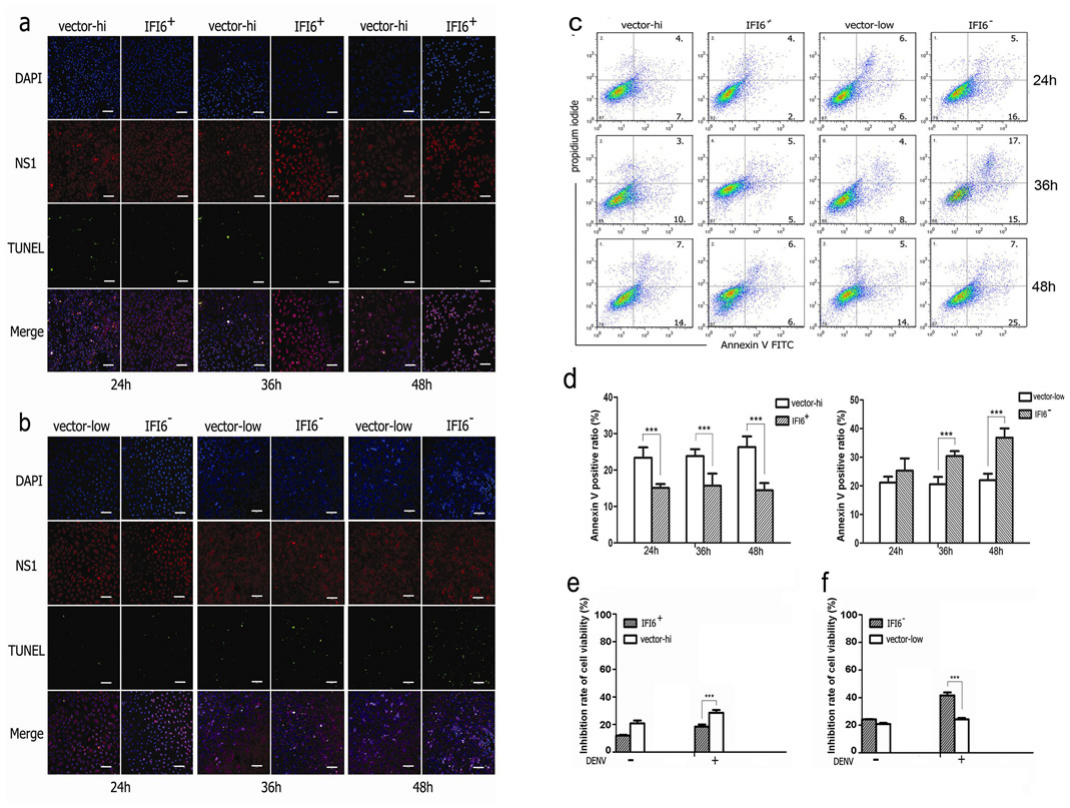
IFI6 inhibits apoptosis of VECs induced by DENV2. (a and b) Apoptosis of IFI6+/+ cells, IFI6-/- cells and vector cells were detected using TUNEL assay after 24, 36, and 48 hrs post DENV2 infection.(c) Apoptosis of IFI6+/+ cells, IFI6-/- cells and vector cells were detected using AnnexinV-FITC/PI labeling flow cytometry after 24, 36, and 48 hrs post DENV2 infection.(d) Analysis of AnnexinV positive cell ratio.(e and f) Cell viability was determined by MTT. ***P<0.01.

A number of studies had already shown that DENV2 could induce apoptosis in VECs [23–25]. Thus we explored the potentially important role that IFI6 might play in this pathological process of programmed cell death. With the aid of fluorescence confocal microscopy, we observed that the non-structual 1 protein (NS1) of DENV2 appeared in more than 90% of the recombinant VECs after 24 hrs post DENV2 incubation. Evidence of apoptosis was found scattered within the infected cells. However, there seemed to be almost no DNA fragments at the early stages of infection in IFI6+/+ cells, which maintained a lower level of apoptosis as compared with the vector-high cells (Fig 3a). On the contrary, DNA fragments had increased in IFI6-/- cells as compared to vector-low cells at optional time points, especially at 48 hrs after infection (Fig 3b).

Annexin V/propidium iodide (PI) staining and flow cytometry assays were also performed to distinguish the early-stages of apoptosis. The results demonstrated that after 24 to 48 hrs following DENV2 infection, Annexin V positive cells were constantly lower in IFI6+/+ cells than in the corresponding vector-high cells (left panels in Fig 3c and 3d). Concomitantly, this observation appeared much more frequent in IFI6-/- cells as compared to the vector-low cells, with statistical differences seen at 36 and 48 hrs post DENV2 infection (right panels in Fig 3c and 3d). We also enumerated cell viability by MTT assay (Fig 3e and 3f). Viability of IFI6+/+ cells was higher than vector high cells and viability of IFI6-/- cells was lower than vector-low cells. These data indicated that IFI6 may play important roles in apoptosis process induced by DENV2 infection

### 3.4 IFI6 inhibits DENV2-induced VECs apoptosis via caspase-dependent pathway

**Fig 4:**
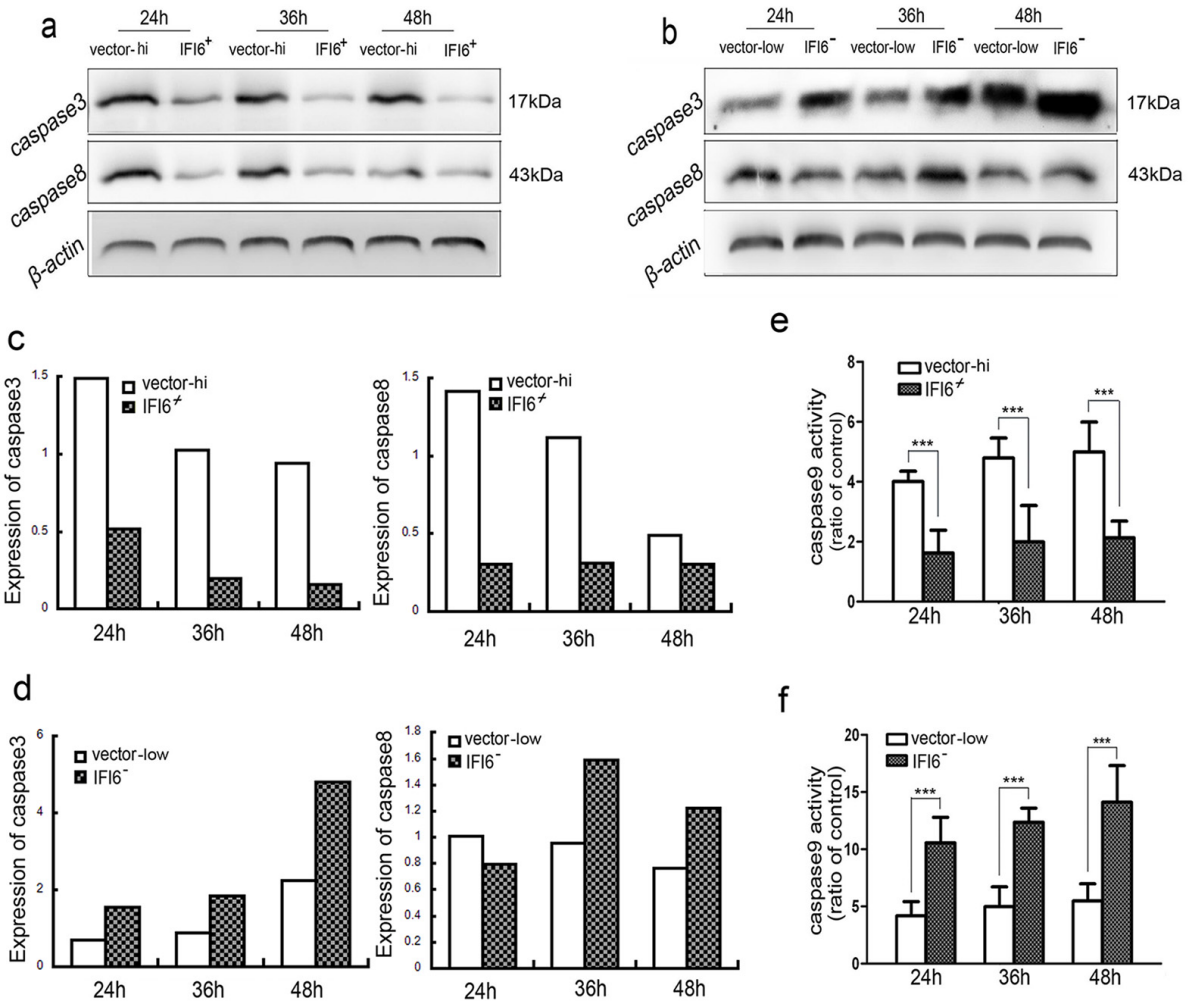
IFI6 ihibits the expression of caspase-3/caspase-8 and the activity of caspase-9. (a and b) Immunoblotting was used to detect the expressions of caspase-3 and caspase-8 in IFI6+/+ cell lines and IFI6-/- cell lines that were infected by DENV2 (MOI = 4) for 24, 36 and 48 hrs, respectively. (c and d) The activity of caspase-9 was analyzed using a colorimetric assay. The results shown were from three independent experiments. ***P<0.01.

To determine whether intrinsic or extrinsic pathways were activated during apoptosis induced by DENV2 infection, we detected protein levels of caspase-3 and 8 using immunoblotting and anzyme activity of caspase-9 using colorimetry. As shown in Fig 4a, stronger inhibition of cleaved caspase-3 occurred time-dependently in IFI6+/+ cells as compared with vector-high cells. By stark contrast, an extremely increased level of cleaved caspase-3 was observed in IFI6-/- cells as compared with vector-low cells (Fig 4b). Caspase-8 was expressed at much lower levels at the early stages of DENV2 infection in IFI6+/+ cells as compared with vector-high cells, but there was no marked difference at 48 hrs post-infection. Additionally, increased tendency of caspase-8 activation was also inconspicuous in vector-low cells (Fig 4a and 4b). However, consistent lower activity of caspase-9 was observed in IFI6+/+ cells as compared with vector-high cells (Fig 4c) while much higher activity of caspase-9 was observed in IFI6-/- cells as compared with vector-low cells (Fig 4d).

### 3.5 Mitochondria participates the apoptotic progress induced by DENV2 and IFI6 regulates mitochondrial function

**Fig 5:**
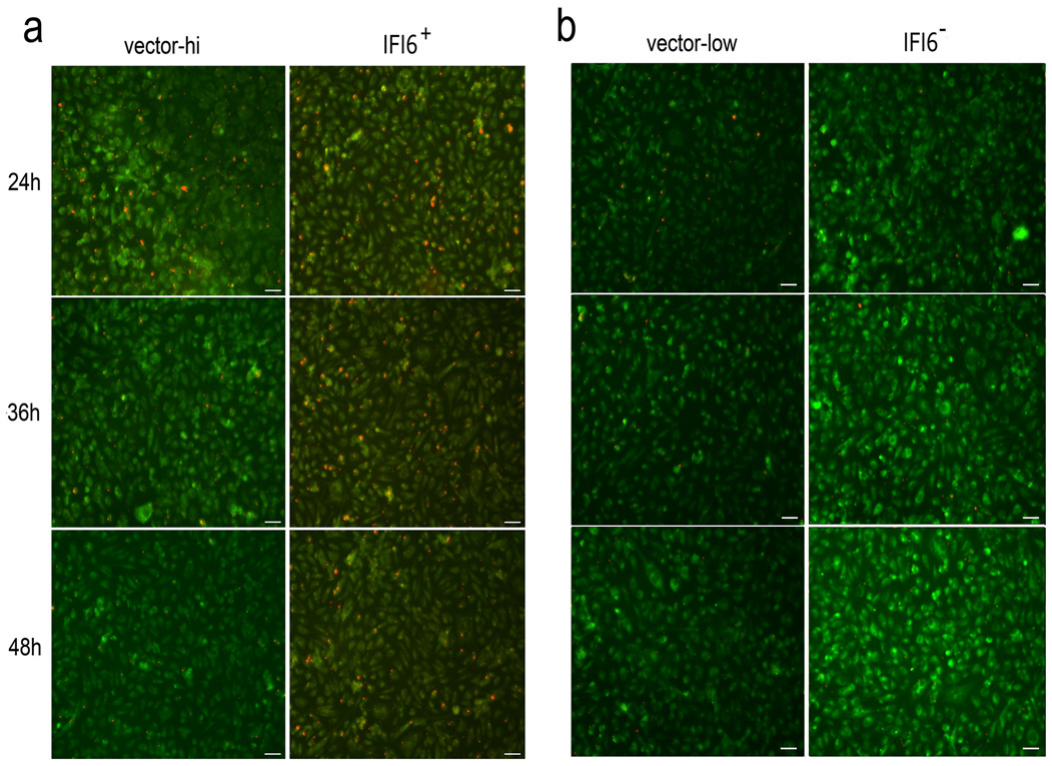
Changes in mitochondrial membrane potential (Δψm) in infected recombinant VECs. Δψm was measured by fluorescence microscopy in vector-hi/IFI6+/+ cells (a) and vector-low/IFI6-/- cells (b) stained by JC-1 after 24, 36 and 48 hrs post DENV2 infection. Scale bar = 50 μm.

It has been established that caspase-9 plays an important role in the intrinsic apoptosis pathway [26], which is mediated by activation of mitochondria. We assayed alterations in the mitochondrial membrane potential (Δψm) in situ by a cationic and voltage-sensitive vital fluorochrome, called JC-1. The lipid dye emits differential fluorescence that is depended on different polymerization forms. In healthy mitochondria with normal Δψm, JC-1 always transfers into the mitochondria and aggregates to a polymer, and emits red fluorescent signals. On damage to the mitochondria, there is depolarization of Δψm, and JC-1 is released to the cytoplasm, where the fluorescence changes to a green signal. As shown in Fig 5, the continued decline of Δψm in both vector cell lines was conspicuous from 24 hrs to 48 hrs after DENV2 infection. The infected IFI6+/+ cells displayed a stable Δψ_m_ as shown by a sustained aggregation of the red JC-1 fluorescence over periods of time (Fig 5a). By contrast, IFI6-/- cells appeared to be more sensitive to DENV2, which was indicated by complete vanishing of the red fluorescent signal at early-stages post-infection, and the ongoing enhanced green fluorescence of the JC-1 aggregates (Fig 5b).

### 3.6 IFI6 regulates apoptosis-related proteins Bcl-2/Bax and XAF1

**Fig 6:**
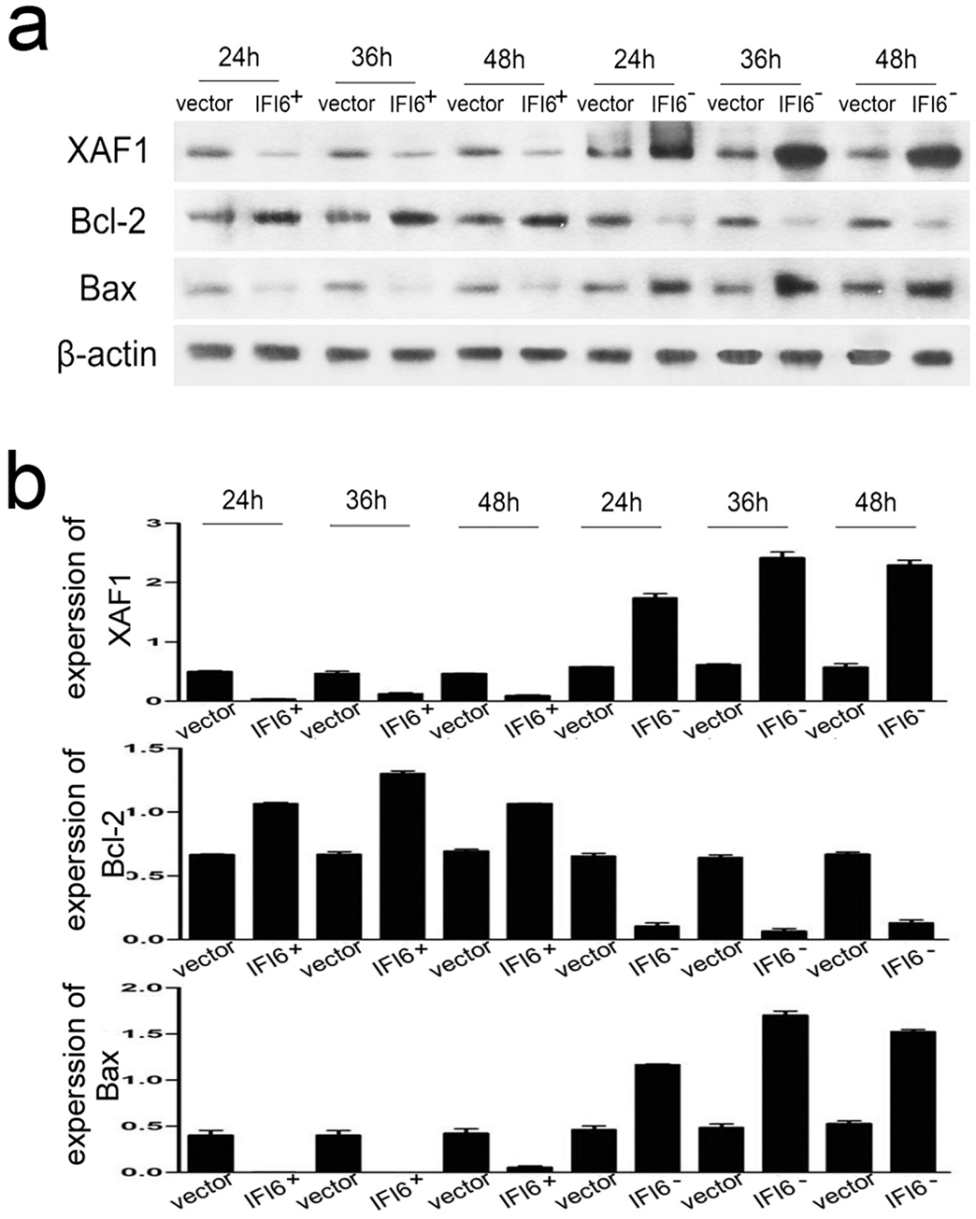
IFI6 influences the expressions of XAF1, bcl-2 and bax. (a) Recombinant cells were infected by DENV2 (MOI = 4) for 24, 36 and 48 hrs. Immunoblotting was used to detect the expression of XAF1, Bcl-2 and Bax in IFI6 over-expression or knock-down cell lines. (b) Quantitative data in panel a. The results shown were from three independent experiments.

To verify whether the change in Δψ_m_ was associated with a balance in the expression of pro-apoptotic and anti-apototic proteins Bcl-2/Bax in the mitochondria or in other subcellular structures, we performed immunoblotting assays. The infected IFI6+/+ cells provoked a much higher level of Bcl-2, but a much lower level of Bax and the nuclear protein XAF1 as compared with the vector-high cells at the outset of virus challenge (Fig 6a). Additionally, Bcl-2 expression was lower in IFI6-/- cells, while expressions of Bax and XAF1 were both higher than those of vector-low cells at all time-points measured (Fig 6b).

## Discussion

IFNα/β is elicited early in DENV2 infected cells, which had been long recognized in clinical cases [27]. IFNα/β serves as an early barrier to DENV infection, and is specifically indispensable for restricting the initial replication or spreading of DENV [5]. Therefore, IFI6, which is induced by IFNα/β, may play a critical role in combating viral infection and maintaining cell survival. In our previous study, we observed enhanced IFI6 expression by differential display microarray analysis of DENV infected VECs [19]. In current study we also detected mRNA expression of IFI6 by qRT-PCR at 48 hrs post-infection in primary HUVECs, and found that IFI6 expression was dramatically increased in infected HUVECs. These observations were confirmed by immunoblotting analysis of protein levels to find that IFI6 level in infected EA.hy926 cells exceeded that of uninfected cells.

For the last decade, IFI6 was generally regarded as a pro-survival gene since it is expressed at high levels in gastric cancer, colorectal cancer, breast cancer, myeloma, tongue squamous cell carcinoma, and in psoriasis [14, 28–32]. To know more about the role played by IFI6 in DENV infected VECs, we are committed to construct recombinant cells displaying stable over-expression or knock-down of IFI6. But HUVECs are not sensitive to the transfection,so we choose EA.hy926 cells which exhibited endothelial cell phenotype to construct the recombinant cells. We found that IFI6+/+ VECs exhibited enhanced cell viability and resistance to DENV2-induced apoptosis. On the contrary, cell death was significantly accelerated and aggravated in IFI6-/- cells. This finding is in agreement with reports of the role of IFI6 in cancer cell survival and death [14]. IFI6 also serves as a potential molecular mechanism that tumor cells might employ to resist chemotherapeutic drugs, including cycloheximide (CHX) or 5-fluorouracil (5-FU) because its expression is potently enhanced in multidrug-resistant malignant cells and attenuates apoptosis following anti-tumor therapy [10].

Many investigators have shown that DENV2 might induce apoptosis by extrinsic and intrinsic pathways [33]. Caspase-3 activation is an important event in cell death. Here, we observed impaired functional expression and activation of caspase-3 in infected IFI6+/+ cells and remarkable expression in the corresponding IFI6-/- cells. Both caspase-9 and caspase-8 are generally considered the apical caspases in the intrinsic and extrinsic pathways respectively [34]. After incubation with DENV2, both caspase-8 protein and caspase-9 activity were down-regulated in IFI6+/+ cells. However, only the latter maintained the upward trend in functional activation in IFI6-/- VECs. Thus, it appears that the intrinsic-dependent pathway is more clearly targeted by expression of the IFI6 gene following DENV infection, and not necessarily the extrinsic pathway *per se*.

Both caspase-9, and caspase-3 activation are always engaged following altered integrity of the mitochondrial membrane potential (Δψ(m)), and both caspases are major elements of the mitochondrial pathway of apoptosis [35]. Compelling evidence obtained from the JC-1 staining assay revealed that IFI6 stabilized Δψ(m), and delayed mitochondrial dysfunction of the infected VECs. To the best of our knowledge, there is only study available from work that investigated gastric cancer cells that report the location of *IFI6* in the mitochondria, and the anti-apoptotic function is expressed by inhibiting depolarization of Δψ(m) and interacting with calcium and integrin-binding protein [16]. Similarly, our study showed that the Bcl-2/Bax rheostat participated in the anti-apoptotic process of IFI6.

XAF1, a well-known apoptotic protein, is induced by both TNF-α and IFN [12]. From our previous study, we found that expression of XAF1 was highly expressed in DENV2 infected VECs, with a peak occurrence at later stages (48 hrs) of viral challenge [19]. Following this enhanced expression of XAF1, as much as 28% of the cells had entered irreversible apoptosis by 72 hrs post-infection [36], at which point the balance of survival had been disrupted. In this study we found that during DENV2 infection, XAF1 was down-regulated by over-expressed IFI6. By contrast, XAF1 expression was increased in VECs in which IFI6 was knocked down.

## Conclusion

**Fig 7:**
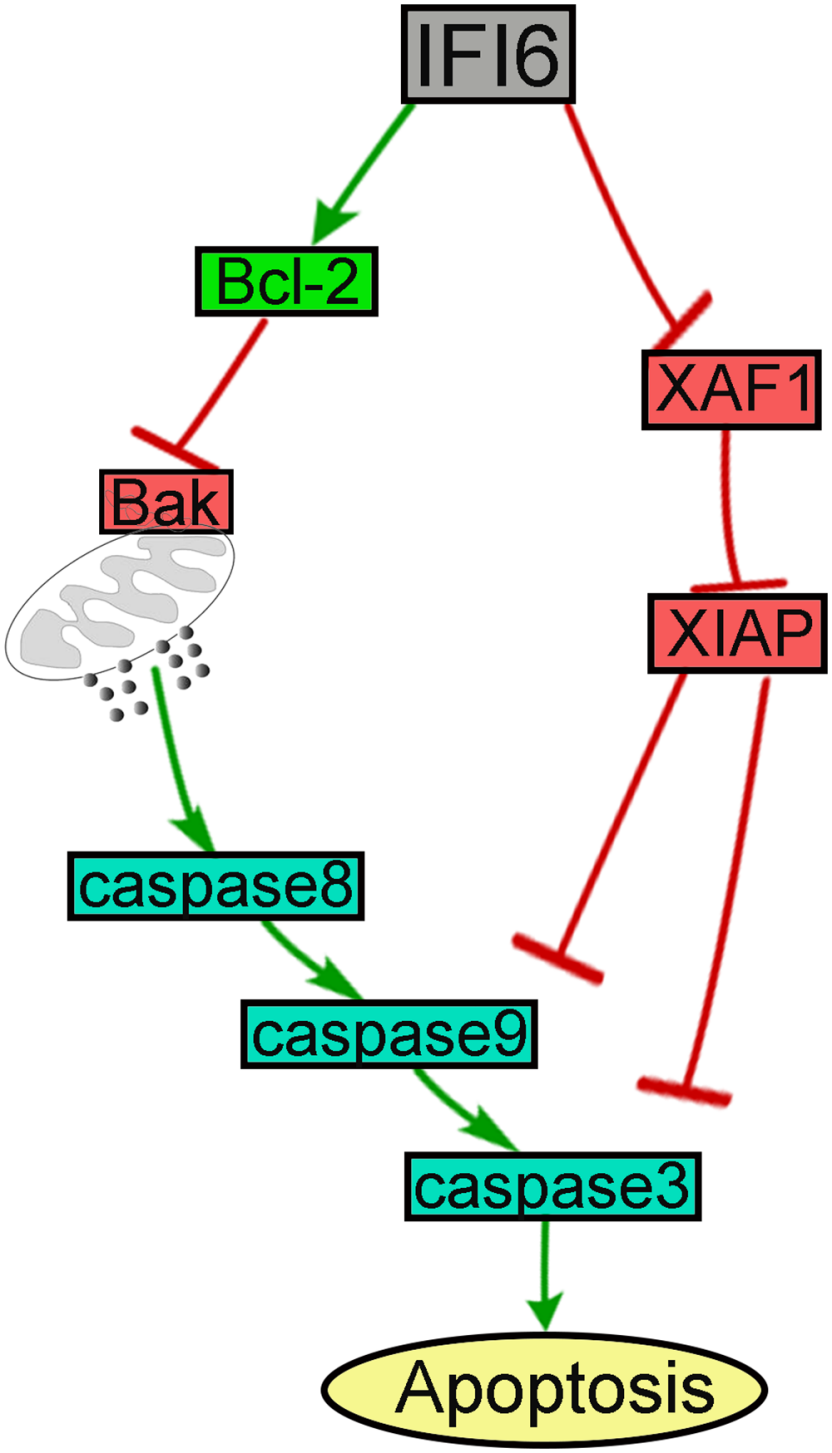
The schematic be used to summarize the signal transduction pathway elicited in ECs by dengue.

Our results indicated that IFI6 might play a critical role in counteracting apoptosis during DENV infection. However, the underlying mechanisms of IFI6 function involves apoptosis-related proteins expressed differently and their effects on caspases(Fig 7),which needed further investigation. At present we speculate that IFI6 might function as a protective gene during DENV infection in humans, it might represent a potential new therapeutic target in the setting of DHF/DSS.
