# Correction: IFI6 Inhibits Apoptosis via Mitochondrial-Dependent Pathway in Dengue Virus 2 Infected Vascular Endothelial Cells

**DOI:** 10.1371/journal.pone.0138896

**Published:** 2015-09-22

**Authors:** Yiming Qi, Ying Li, Yingke Zhang, Lin Zhang, Zilian Wang, Xuzhi Zhang, Lian Gui, Junqi Huang

1. There are errors in the author affiliations. The affiliations should appear as shown here:

Yiming Qi^1,2,4,5^, Ying Li^1,2,4,5^, Yingke Zhang^1,2,4,5^, Lin Zhang^1,2,4,5^, Zilian Wang^3^, Xuzhi Zhang^1,2,4,5^, Lian Gui^1,2,4,5^, Junqi Huang^1,2,4,5*^.

**1** Guangdong Provincial Key Laboratory of Organ Donation and Transplant Immunology, The First Affiliated Hospital, Sun Yat-sen University, Guangzhou, PR China, **2** Organ Transplant Center, The First Affiliated Hospital, Sun Yat-sen University, Guangzhou, PR China, **3** Department of Obstetrics and Gynecology, The First Affiliated Hospital, Sun Yat-sen University, Guangzhou, PR China, **4** Key Laboratory of Tropical Diseases Control, Ministry of Education, Guangzhou, PR China, **5** Institute of Immunology, Zhongshan School of Medicine, Sun Yat-sen University, Guangzhou, PR China

2. There are errors in the Funding section. The correct funding information is as follows: This work was supported by the National Natural Science Foundation of China (no.30872350 and no.31370870) http://isis.nsfc.gov.cn; Natural Science Foundation of Guangdong Province (no.S81510008901000017, no.S2012010009050 and no.S2013020013000) http://gdsf.gdstc.gov.cn; Guangdong Province Scientific Technology Project (no. 2010B050700008, no. 2011B040300022 and no.2013A020229003) http://www.gdstc.gov.cn; Guangzhou City Scientific Technology Project (no.11C32060749, no. 2011J4100084 and no. 2008Z1-E221) http://apply.gysi.gov.cn; The Fundamental Research Funds for the Central Universities (no. 10YKPY31) http://mso.sysu.edu.cn; Guangdong Provicial Key Laboratory Construction Projection on Organ Donation and Transplant Immunology (no.2013A061401007) http://www.gdstc.gov.cn. The funders had no role in study design, data collection and analysis, decision to publish, or preparation of the manuscript.
